# Combined Application of Quantitative Susceptibility Mapping and Diffusion Kurtosis Imaging Techniques to Investigate the Effect of Iron Deposition on Microstructural Changes in the Brain in Parkinson’s Disease

**DOI:** 10.3389/fnagi.2022.792778

**Published:** 2022-03-15

**Authors:** Lin Yang, Yan Cheng, Yongyan Sun, Yinghua Xuan, Jianping Niu, Jitian Guan, Yunjie Rong, Yanlong Jia, Zerui Zhuang, Gen Yan, Renhua Wu

**Affiliations:** ^1^Department of Radiology, The Second Affiliated Hospital of Xiamen Medical College, Xiamen, China; ^2^Department of Radiology, The Second Affiliated Hospital, Medical College of Shantou University, Shantou, China; ^3^Department of Pharmacy, Guangdong Second Provincial General Hospital, Zhuhai Hospital, Zhuhai, China; ^4^Department of Basic Medicine, Xiamen Medical College, Xiamen, China; ^5^Department of Neurology, The Second Affiliated Hospital of Xiamen Medical College, Xiamen, China; ^6^Department of Ultrasound, Foshan Women and Children’s Hospital Affiliated to Southern Medical University, Foshan, China; ^7^Department of Neurosurgery, Sun Yat-sen Memorial Hospital, Sun Yat-sen University, Guangzhou, China; ^8^Guangdong Provincial Key Laboratory for Breast Cancer Diagnosis and Treatment, Cancer Hospital of Shantou University Medical College, Shantou, China

**Keywords:** Parkinson’s disease (PD), quantitative susceptibility mapping (QSM), diffusion kurtosis imaging (DKI), iron content, microstructure

## Abstract

**Objectives:**

Brain iron deposition and microstructural changes in brain tissue are associated with Parkinson’s disease (PD). However, the correlation between these factors in Parkinson’s disease has been little studied. This study aimed to use quantitative susceptibility mapping combined with diffusion kurtosis imaging to investigate the effects of iron deposition on microstructural tissue alterations in the brain.

**Methods:**

Quantitative susceptibility mapping and diffusion kurtosis imaging were performed on 24 patients with early PD, 13 patients with advanced PD, and 25 healthy controls. The mean values of magnetic susceptibility and diffusion kurtosis were calculated for the bilateral substantia nigra, red nucleus, putamen, globus pallidus, and caudate nucleus, and compared between the groups. Correlation analyses between the diffusion kurtosis of each nucleus and its magnetic susceptibility parameters in PD patients and healthy controls were performed.

**Results:**

The study found a significant increase in iron deposition in the substantia nigra, red nucleus, putamen and globus pallidus, bilaterally, in patients with PD. Mean kurtosis values were increased in the substantia nigra but decreased in the globus pallidus; axial kurtosis values were decreased in both the substantia nigra and red nucleus; radial kurtosis values were increased in the substantia nigra but showed an opposite trend in the globus pallidus and caudate nucleus. In the substantia nigra of patients with PD, magnetic susceptibility was positively correlated with mean and radial kurtosis values, and negatively correlated with axial kurtosis. None of these correlations were significantly different in the control group. In the putamen, magnetic susceptibility was positively correlated with mean, axial, and radial kurtosis only in patients with advanced-stage PD.

**Conclusion:**

Our study provides new evidence for brain iron content and microstructural alterations in patients with PD. Iron deposition may be a common mechanism for microstructural alterations in the substantia nigra and putamen of patients with PD. Tracking the dynamic changes in iron content and microstructure throughout the course of PD will help us to better understand the dynamics of iron metabolism and microstructural alterations in the pathogenesis of PD and to develop new approaches to monitor and treat PD.

## Introduction

Parkinson’s disease (PD) is characterized clinically by rest tremor, bradykinesia, rigidity, and postural instability. The main pathological change in PD is dopaminergic neuron degenerative death in the nigrostriatal system due to iron deposition (Dexter et al., 1987; Jin et al., 2011; Kalia and Lang, 2015; Xu et al., 2021). The link between excessive iron deposition and the pathophysiology of PD has been shown in recent studies that revealed that ferrous iron promotes both oxidative stress and α-synuclein aggregation (Wolozin and Golts, 2002; Barnham et al., 2004). Several studies have demonstrated that a variety of microstructural changes occur in the brains of patients with PD (Taylor et al., 2018). In addition to the loss of dopaminergic neurons, there is an accumulation of Lewy bodies (LBs) and Lewy neurites (LNs) (Covell et al., 2017; Ghosh et al., 2017), neuroinflammation (Kumar et al., 2012), and glial cell proliferation (Batassini et al., 2015). Non-invasive understanding of pathological changes by imaging is important for early detection of the disease and guidance of effective treatment. Although iron deposition can lead to microstructural changes in the gray matter nuclei, studies investigating associations between brain iron deposition and microstructural changes in the brain in PD are rare and the associations have not been evaluated using imaging techniques. Quantitative susceptibility mapping (QSM) is a new post-processing technique that provides a robust magnetization measurement that correlates significantly with brain iron content, enabling quantitative tissue magnetization measurement (He et al., 2015; Du et al., 2016). This approach detects magnetic tissue properties more sensitively than traditional quantitative-based iron imaging techniques (R2, R2*, and R2’) and has been used to identify several human brain substructures that are partially indistinguishable when using Gradient Echo (GRE)-based comparisons (Wieler et al., 2015; Guan et al., 2017b).

Diffusion kurtosis imaging (DKI) is a state-of-the-art method for quantifying non-Gaussian water diffusion (Jensen et al., 2005; Coutu et al., 2014; Filli et al., 2014). An alternative, diffusion tensor imaging, does not consider the isotropic nature of gray matter structure (Pierpaoli and Basser, 1996). Therefore, DKI is better suited for quantifying subtle pathological changes in gray matter than diffusion tensor imaging (Jensen and Helpern, 2010). By measuring direction-specific kurtosis, DKI reflects the complexity of neural tissue in normal, developmental, and pathological states. Mean kurtosis (MK), axial kurtosis (Ka), and radial kurtosis (Kr) are direction-specific kurtosis parameters. It has been suggested that Kr decrease is associated with demyelination, Ka changes reflect axonal degeneration (Cheung et al., 2009), and increases in MK may indicate injury-related microglial proliferation and increased axonal bead granulation (Weber et al., 2015).

Magnetic susceptibility (MS) (Li et al., 2018; Uchida et al., 2019, 2020) and diffusion kurtosis parameters (Wang et al., 2011; Kamagata et al., 2017; Guan et al., 2019) have been shown in past studies to reflect brain iron deposition levels and brain tissue microstructural changes, respectively, in patients with PD. The substantia nigra (SN), red nucleus (RN), and striatum are the main nuclei involved in PD. We hypothesized that there may be a correlation between excessive iron deposition in these regions of the brain and alterations in apparent diffusion kurtosis in patients with PD. To our knowledge, few previous reports have combined QSM and DKI in the evaluation of PD. Herein, we aimed to jointly apply QSM and DKI techniques to investigate microstructural changes in the gray matter nuclei, due to iron deposition, and identify the specific features of observed changes. Enhancing our understanding of correlations between the findings of magnetic susceptibility and diffusion kurtosis may improve our knowledge of pathological changes in PD and their effects on disease activity and contribute to early detection and treatment.

## Materials and Methods

### Subjects

All examinations in this study were performed with the written consent of each participant, and the study was approved by the Ethics Department of the Second Affiliated Hospital of Xiamen Medical College. All processes were conducted in accordance with the principles of the Declaration of Helsinki. In the study, we prospectively assessed 37 patients with PD who attended the Department of Neurology at the hospital between January 2019 and December 2020. All patients were diagnosed with PD by a movement disorder neurologist (Dr. Niu, with more than 30 years of experience), underwent MRI, and met British Parkinson’s Disease Association Brain Bank criteria. The following exclusion criteria were applied: atypical or secondary PD, history of other neurological disease e.g., severe head trauma or stroke, poor image quality, or general MRI scan contraindications. All patients were older than 50 years, right-handed, and underwent a thorough neurological examination. Patients and their families provided detailed information on the onset, course, and evolution of PD. Hoehn-Yahr (HandY) stage and Unified Parkinson’s Disease Rating Scale (UPDRS) were used to assess disease severity and motor function (Greffard et al., 2006).

Patients were further categorized into early-stage PD (ESPD) (HandY stage ≤ 2.5; 12 men, 12 women; mean age, 63.0 ± 7.4 years) and advanced stage PD (ASPD) (HandY stage ≥ 3; 8 men, 5 women; mean age, 71.1 ± 7.5 years) groups. PD duration was defined as the time difference between first motor symptom development and the study examination date. The mental status of all patients was assessed using the Mini-mental State Examination (MMSE) and the Montreal Cognitive Assessment (MoCA) scores (Folstein et al., 1975).

Since both methods have unique characteristics, we conducted two PD tests for each patient (Pinto et al., 2019). Clinical evaluation and MRI imaging were performed at least 12 h after discontinuation of all anti-PD medications. Twenty-five age- and sex-matched healthy control (HC) participants (10 men, 15 women; mean age, 67.0 ± 9.3 years) were recruited from a medical examination center. HC participants met the following inclusion criteria: no history of neuropsychiatric or neurodegenerative disease; no white matter damage, such as epilepsy, multiple sclerosis, tumors, trauma, cranial arteritis, or encephalitis; no history of alcohol dependence or other psychoactive substance abuse; and MMSE score > 28 points.

### Imaging Protocol

All participants underwent MRI examinations on a 3 T scanner (Discovery MR750, GE Healthcare, Milwaukee, WI, United States) equipped with an eight-channel phased array receiver coil. Participants were instructed to relax and avoid any motion during the test. Noise-proof earplugs and foam pads were applied to minimize acoustic scanner noise and motion artifacts, respectively. Auto shimming was employed at the start of each scan to ensure uniformity of the static magnetic field. Before QSM and DKI imaging, routine images, including T1-weighted imaging, T2-weighted imaging, fluid-attenuated inversion recovery (FLAIR) images, and diffusion-weighted imaging (DWI), were acquired. They were used to confirm the absence of structural abnormalities, and to exclude secondary Parkinson’s syndrome caused by severe vascular disease, multisystem atrophy, trauma, or encephalitis.

Diffusion kurtosis imaging images were obtained using a single-shot spin-echo echo-planar imaging (SE-EPI) sequence with the following parameters: repetition time (TR) = 3,000 ms; echo time (TE) = 60 ms; slice thickness/gap = 5/1.5 mm; field of view (FOV) = 240 × 240 mm; matrix size = 128 × 128; number of excitations (NEX) = 1; *b*-values = 0, 1,000 (30 directions), and 2,000 (30 directions) s/mm^2^; number of slices = 15; total scan time = 3 min 2 s. QSM source images, including magnitude and phase images, were obtained using a three-dimensional multi-echo fast spoiled gradient recalled echo (FSPGR) sequence with the following parameters: TR = 23.7 ms; TE = 3.4/5.8/8.1/10.5/12.8/15.2/17.5/19.9 ms; flip angle = 12°; slice thickness/gap = 1/0 mm; FOV = 256 × 256 mm; matrix size = 256 × 256; NEX = 1; number of slices = 140; total acquisition time = 5 min 1 s. Two experienced neuroradiologists provided diagnostic support. All sequences were acquired in the axial plane parallel to the anterior commissure-posterior commissure (AC-PC) line. All images were carefully reviewed after scanning to ensure image quality, and poor image quality due to motion artifacts prompted rescanning.

### Image Analyses and Region-of-Interest Selection

Raw DKI and QSM data were transferred to the Advantage Workstation 4.6 (GE Healthcare) and post-processed by the FuncTool software. Specifically, DKI parameter maps, including MK, Ka, and Kr, were calculated by using the following equation (Jensen et al., 2005):


$$\ln\left[S_{\left(n,b\right)}/S_{0}\right]=-b\,\sum_{i=1}^{3}\sum_{j=1}^{3}n_{i}\,n_{j}\,D_{i\,j}+\frac{1}{6}\,b^{2}\,D^{-2}\,\sum_{i=1}^{3}\sum_{j=1}^{3}\sum_{k=1}^{3}\sum_{l=1}^{3}n_{i}\,n_{j}\,n_{k}\,n_{l}\,W_{i\,j\,k\,l}$$

$$\ln\left[S_{\left(n,b\right)}/S_{0}\right]=$$

$$-b\,\sum_{i=1}^{3}\sum_{j=1}^{3}n_{i}\,n_{j}\,D_{i\,j}$$

$$+\frac{1}{6}\,b^{2}\,D^{-2}\,\sum_{i=1}^{3}\sum_{j=1}^{3}\sum_{k=1}^{3}\sum_{l=1}^{3}n_{i}\,n_{j}\,n_{k}\,n_{l}\,W_{i\,j\,k\,l}$$

*S*_(_*_*n,b*_*_)_ denotes the diffusion encoding direction *n* and the diffusion signal intensity of the diffusion-weighted *b*-value, *S*_0_ denotes the diffusion signal intensity of *b*_0_, and *D*_*ij*_ and *W*_*ijkl*_ represent the components of the diffusion tensor and the diffusion kurtosis tensor, respectively. We have also used this DKI analysis method in our earlier studies (Zheng et al., 2017; Yang et al., 2021). Multi-echo QSM data were processed by Laplacian-based phase unwrapping, and V-SHARP background field removal (Li et al., 2014b), and improved the sparse linear equation and least squares (iLSQR) method (Li et al., 2015b) to generate MS maps, based on the images of the last three echoes.

Regions of interest (ROIs) were delineated three times manually by two independent, double-blinded neuroradiologists with B0 images as references, and values were recorded each time to reduce offset errors (Supplementary Figure 1). The average of the six time delineation for the ROI values was taken as the final value. Each nucleus side was recorded as a separate sample. The mean parametric values of the bilateral SN, RN, globus pallidus (GP), putamen, and caudate nuclei were used for further analysis. The intraclass correlation coefficient (ICC) was used to assess the agreement between the two neuroradiologists for the MS, MK, Ka, and Kr measurements (Landis and Koch, 1977). Usually, ICC values > 0.75 are considered good correlation. To minimize deviation due to partial volume effects, the following criteria were applied: (1) choose the clear boundary and the largest display area of each nucleus to outline the ROI, and carefully avoid blood vessels and cerebrospinal fluid; (2) when delineating the nucleus boundary, move one pixel inward to ensure that the ROI is within the range of the nucleus.

### Statistical Analyses

All data were analyzed using SPSS Statistics Package, version 19.0 (IBM Corporation, New York, NY, United States). The Kolmogorov–Smirnov test was used to confirm the normal distribution of data. One-way analysis of variance (ANOVA) or unpaired *t*-tests were used to compare demographic information and clinical characteristics among the groups. Average MS values and corresponding 95% confidence intervals of QSM imaging data were calculated for each region, along with MK, Ka, and Kr values from DKI. One-way ANOVA followed by false discovery rate (FDR) correction, as described by the Benjamini–Hochberg method, were used to compare the differences in MS, MK, Ka, and Kr values using the R software package (R for Windows v. 4.0.3)^1^ in patients with different stages of PD to HCs. For intergroup comparisons of non-normally distributed data, the non-parametric Kruskal–Wallis test was used. Pearson correlation analyses were used to investigate the relationship between the MS of the nucleus and the MK, Ka, and Kr values in patients with PD and HCs. Nuclei with good correlations in prior assessments were further divided to investigate correlations with different PD stages. We performed an FDR correction for multiple correlation tests. Finally, the associations between the QSM and DKI parameters and disease severity in the SN were examined using the Pearson correlation test, with adjustments for age and sex effects. For all analyses, values of *P* < 0.05 were considered significant.

## Results

### Demographics and Neuropsychiatric Assessment

The demographic and clinical characteristics of the participants are shown in Table 1. Although the proportion of males with PD was higher than that of females, consistent with the epidemiological characteristics of PD, no significant sex differences were observed among the groups. Further, no significant between-group differences regarding age, or MMSE, MoCA, or UPDRS Part I and Part IV scores were observed. The disease duration of patients with ASPD was significantly longer than that of ESPD (*p* < 0.001). Meanwhile, the UPDRS-total, UPDRS-II, UPDRS-III scores, and the HandY stage of patients with ASPD were significantly greater than those of ESPD (*p* < 0.05).

**TABLE 1:** Demographics of patients with Parkinson’s disease and healthy controls.

Variable	Normal (*N* = 25)	ESPD (*N* = 24)	ASPD (N = 13)	*p-*Value
Male sex, *N* (%)	10.0 (42.7)	12.0 (50.0)	8.0 (61.5)	0.443^a^
Age, years [Mean (SD)]	67.0 (9.3)	63.0 (7.4)	71.1 (7.5)	0.128^b^
Disease duration, years [Mean (SD)]	–	3.5 (5.7)	9.0 (7.3)	**<0.001^c^**
MMSE, [Mean (SD)]		22.4 (5.9)	18.9 (6.0)	0.225^c^
MoCA, [Mean (SD)]		18.4 (6.6)	16.0 (7.5)	0.460^c^
Hoehn-Yahr stage, Mean (SD)	–	1.6 (0.5)	3.7 (0.8)	**<0.001^c^**
UPDRS score, Mean (SD)	–			
Total	–	29.4 (12.0)	44.6 (12.7)	**0.010^c^**
Part I	–	2.1 (1.7)	2.1 (2.0)	0.798^c^
Part II	–	8.8 (4.8)	15.0 (5.3)	**0.010^c^**
Part III	–	17.7 (7.8)	25.8 (5.9)	**0.019^c^**
Part IV^d^	–	1.9 (1.8)	1.8 (2.1)	0.904^c^

*ESPD, early-stage Parkinson’s disease; ASPD, advanced-stage Parkinson’s disease; SD, standard deviation; UPDRS, unified Parkinson’s disease rating scale; MMSE, mini-mental state examination; MoCA, montreal cognitive assessment. Bold values indicate statistically significant differences.*

*^a^According to Pearson chi-square test.*

*^b^According to One-Way ANOVA.*

*^c^According to Unpaired t-test.*

*^d^Provided for patients using levodopa.*

### Group Differences Assessed via Quantitative Susceptibility Mapping and Diffusion Kurtosis Imaging

**FIGURE 1:**
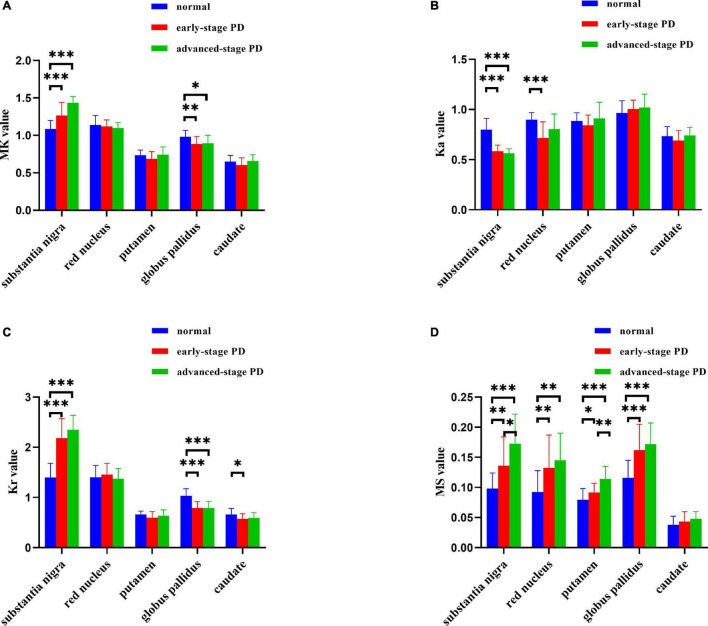
**(A–D)** Histograms depicting intergroup comparisons of imaging parameters in the substantia nigra, red nucleus, globus pallidus, putamen, and caudate nucleus (FDR-corrected). Error bars represent standard errors of the mean (*P < 0.05; ^**^P < 0.01; ^***^P < 0.001). MK, mean kurtosis; Ka, axial kurtosis; Kr, radial kurtosis; MS, magnetic susceptibility. The normal group is shown in blue, the early-stage PD group is shown in red, and the advanced-stage PD group is shown in green.

The results of the ICC analysis of the QSM and DKI parameter values for the left and right ROIs of HCs and patients with PD are shown in Supplementary Table 1. The results showed that the ICC values for both the HC and PD groups were >0.75, so the consistency of measurement was reliable enough to continue with the subsequent statistical analysis. MS and DKI values of deep gray matter nuclei in HCs and patients with PD are presented in Figure 1 and Supplementary Table 2. We found that the MS of the SN in the healthy group was significantly lower than that of the ESPD and ASPD groups (*p* = 0.003 and *p* < 0.001, respectively), indicating that the SNs of patients with PD have greater paramagnetism, and thus, greater iron deposition levels than healthy people. We also found that MK and Kr values in the SN in the PD group were higher than that of the control group, while the Ka of the control group was higher than that of the PD group. The MS of the RN was elevated in patients with different stages of PD compared to HCs (*p* = 0.004 and 0.001, respectively), while Ka in the ESPD group decreased more than in the HCs (*p* < 0.001). Although the MS value of the putamen in patients with ASPD was significantly higher than that of the HCs (*p* < 0.001), diffusion kurtosis did not change significantly. Compared with the HCs, the MS of the GP of ESPD and ASPD patients increased (*p* < 0.001 and *p* < 0.001, respectively), while MK (*p* = 0.004 and 0.02, respectively) and Kr (*p* < 0.001 and *p* < 0.001, respectively) decreased. No significant differences in Ka were found in this experiment. A slight decrease in the Kr of the caudate nucleus in ESPD, compared to HCs, was observed (*p* = 0.02).

### Associations Between Diffusion Kurtosis Imaging and Magnetic Susceptibility Parameters in the Nuclei of Patients With Parkinson’s Disease

**FIGURE 2:**
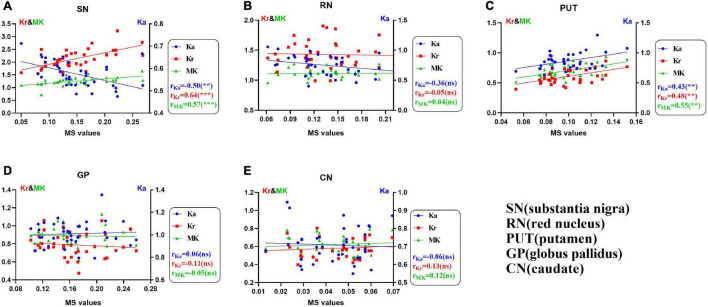
**(A–E)** Univariate correlations of diffusion kurtosis parameters with magnetic susceptibility values in patients with Parkinson’s disease in five nuclei of interest (**P < 0.01; ***P < 0.001; ns, no statistical significance). False discovery rate correction was used for multiple correlations. MK (green, triangles), mean kurtosis; Ka (blue, circles), axial kurtosis; Kr (red, squares), radial kurtosis; MS, magnetic susceptibility.

**FIGURE 3:**
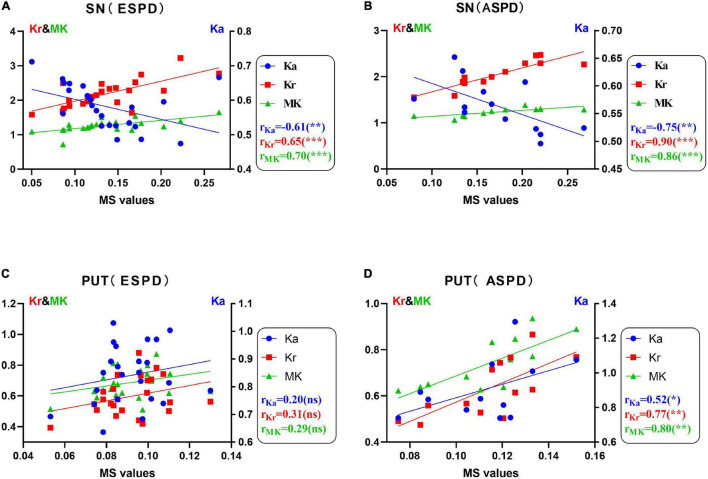
**(A–D)** Correlations between diffusion kurtosis parameters and magnetic susceptibility in the substantia nigra and putamen determined via univariate analysis in patients with different stages of Parkinson’s disease (*P < 0.05; **P < 0.01; ***P < 0.001; ns, no statistical significance). False discovery rate correction was used for multiple correlations. MK (green, triangles), mean kurtosis; Ka (blue, circles), axial kurtosis; Kr (red, squares), radial kurtosis; MS, magnetic susceptibility; ESPD, early-stage Parkinson’s disease; ASPD, advanced-stage Parkinson’s disease.

Figure 2 shows the correlation between the diffusion kurtosis metrics and MS in different brain regions of patients with PD. Figure 3 shows this relationship in the SN and putamen at different stages of disease progression. In the gray matter nuclei of patients with PD, MS, and DKI kurtosis values correlated only in the SN and putamen. Further analysis showed that a correlation between MS and DKI was only observed in the SN in ESPD. In ASPD, there was a correlation between MS and DKI in both the SN and putamen.

### Associations Between Diffusion Kurtosis Imaging and Magnetic Susceptibility Parameters in the Nuclei of Healthy Controls

Supplementary Figure 2 shows the correlation between diffusion kurtosis metrics and MS in different nuclei of the HCs. Our results showed that in HCs, only the Ka of the caudate nucleus was positively correlated with MS. There was no significant correlation between QSM and DKI parameters in the SN, RN, putamen, and GP of HCs.

### Quantitative Susceptibility Mapping and Diffusion Kurtosis Imaging Correlations With Clinical Indices in the Substantia Nigra

The results of our regression analysis are summarized in Supplementary Figure 3. Magnetic susceptibilities and diffusion kurtosis parameters within the SN correlated with the motor and cognitive scores of patients with PD. In the SN of patients with PD, there was a positive correlation between Ka and MMSE (*p* = 0.0305), a positive correlation between Kr and UPDRS III (*p* = 0.0355), and a negative correlation between MK and MMSE and MoCA (*p* = 0.0069 and 0.0233, respectively). MS was positively correlated with HandY staging and UPDRS III (*p* = 0.0268 and 0.0036, respectively), and negatively correlated with MMSE and MoCA scores (*p* = 0.0381 and 0.0399, respectively). The remaining identified correlations were not statistically significant.

### Maps of Quantitative Susceptibility Mapping and Diffusion Kurtosis Imaging at the Midbrain Level

**FIGURE 4:**
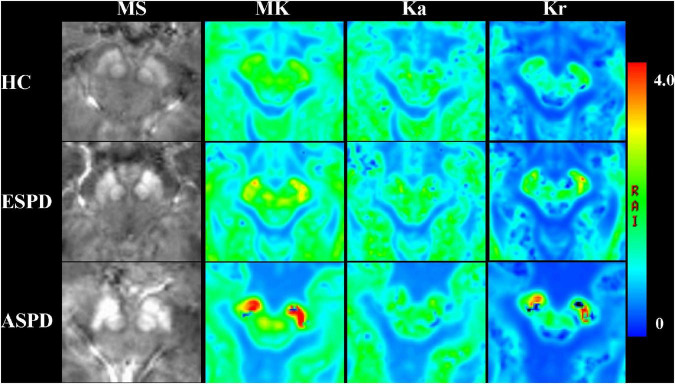
Comparison of magnetic susceptibility and diffusion kurtosis imaging parameter mapping at the midbrain level in healthy participants and patients with Parkinson’s disease. MK, mean kurtosis; Ka, axial kurtosis; Kr, radial kurtosis; MS, magnetic susceptibility; ESPD, early-stage Parkinson’s disease; ASPD, advanced-stage Parkinson’s disease.

Maps of diffusion kurtosis and MS at the midbrain level were successfully constructed. Figure 4 includes representative DKI and QSM maps of the HC and PD groups, where MS demonstrates an increasing signal, which was especially pronounced in the SN of patients with PD. MK and Kr signals in the SN gradually increase throughout disease progression, and the diffusion range correspondingly increases, while Ka decreases. In contrast, only the MS signal increased in the RN, and there was no obvious change in diffusion signal or range.

## Discussion

We used DKI to identify microstructural changes in brain tissue associated with brain iron deposition in patients with PD. We found, for the first time, that microstructural alterations in the SN and putamen have unique characteristics and may be associated with brain iron deposition. In addition, we found that in ESPD, changes in kurtosis correlated with paramagnetism exclusively in the SN. We performed an etiological analysis of these interesting results, which are described below.

### Substantia Nigra

QSM data, shown in Figure 1D, are consistent with previously reported findings, which revealed increased MS values in the SN of patients with PD (Du et al., 2018; Bergsland et al., 2019). MS increases are considered indirect indicators of iron deposition. Abnormal distribution of MS is consistent with LB and LN regions previously identified in the SN of patients with symptomatic PD, corresponding to Braak’s stage III (Braak et al., 2003). Further, patients with PD show abnormalities in DKI parameters (MK, Ka, and Kr), and these differences become more pronounced with disease progression. Patients with PD had elevated MK and Kr values compared to HCs, which is consistent with some (Khairnar et al., 2015; Zhang et al., 2015), but not all, prior reports (Guan et al., 2019). Reported changes in diffusion kurtosis parameter values for PD vary considerably. A major reason for this may be that prior studies did not consider iron deposition levels.

Mean kurtosis elevation in the SN may be due to a combination of several factors. First, following dopaminergic neuronal injury and apoptosis (Zhang et al., 2014), damaged axons may activate major histocompatibility complex class II-positive microglia with phagocytic and trophic functions, leading to elevated tissue structural complexity and kurtosis values (Imamura et al., 2003). Second, loss of dopaminergic neurons in the pars compacta of the SN enhances diffuse heterogeneity due to nigrostriatal pathway deafferentation (Giannelli et al., 2012). Third, LB and LN accumulation in the cytosol of neuronal cells and axons reduces the free diffusion of water, which increases MK (Giannelli et al., 2012).

As iron accumulates, microglia activate to release reactive oxygen species, causing neurotoxicity, and eventually, fluid accumulation in the myelin sheath, causing edema. Because myelin travels directionally, water molecules within myelin do not diffuse freely in each direction, elevating Kr (McGeer and McGeer, 2004; Block et al., 2007).

Axial kurtosis decreases in the SN, which is revealed via DKI in patients with PD and may be due to axonal atrophy in the SN. First, in initial LN accumulation phases in axons, axonal transport (especially reverse axonal transport) is inhibited, which manifests as structural changes of axonal atrophy (Perlson et al., 2010; Millecamps and Julien, 2013). Recently, an *in vivo* high-resolution positron emission tomography study revealed that synaptic density was significantly reduced in the SN, RN, and locus coeruleus in PD (Matuskey et al., 2020). Second, α-synuclein can inhibit axonal branching and growth (Koch et al., 2015).

As shown in Figure 2A, MS was positively correlated with MK and Kr, and negatively correlated with Ka in the SN of patients with PD. In addition, staging analysis correlations revealed that among all gray matter ROIs in patients with PD, correlations between QSM and DKI parameters at an early stage were only observed in the SN, and the association was consistent with that of the overall analysis (Figure 3). The correlations in Figures 2A, 3A,B are consistent with SN parameter trends throughout disease progression, shown in Figures 1, 4. The mechanistic basis of disease progression remains increased levels of iron deposition (Braak et al., 2003).

Our data showed that in the SN of patients with PD, MS correlated positively with HandY staging, and, most significantly, UPDRS III scores, and negatively with cognitive test (MMSE and MoCA) scores, which is consistent with a prior report (He et al., 2015; Langkammer et al., 2016; Guan et al., 2017a,c; Du et al., 2018). It has been demonstrated that iron levels in both the hippocampus and thalamus are higher in patients with PD dementia than in PD and healthy groups (Li et al., 2018). We suggest that the relationship between MS and cognitive scores in Supplementary Figures 3C,D may be a manifestation of iron overload in the SN during progression of brain histopathological stages to the hippocampus (Braak’s stages 5–6) in patients with PD, rather than an indicator that the SN is associated with cognition (Braak et al., 2003).

An interesting phenomenon was observed in the SN of patients with PD, in which Ka was positively correlated with MMSE scores; Kr was positively correlated with UPDRS III scores, and MK was negatively correlated with MMSE and MoCA scores. We hypothesize that some specific microstructural alterations may be associated with clinical symptoms. The presence of a relationship between neuropsychological symptoms and imaging indicators should be investigated at more sites in future studies. Therefore, QSM and DKI parameter abnormalities are clinically significant and likely reflect PD symptom severity.

### Red Nucleus

The RN is composed of dense cells and small myelinated axons with a complex microstructure (Onodera and Hicks, 2009). The ventrolateral magnocellular portion of the RN is directly involved in motor control (Kennedy et al., 1986; Mewes and Cheney, 1994; Rodriguez-Oroz et al., 2008). The RN also contains high levels of iron and may be affected by oxidative stress (Martin et al., 2008). Figure 1D shows that RN MS values in both the ESPD and ASPD groups were higher than that of the HCs. This finding was different from that which was reported by Du et al. (2018) an inconsistency that may have been due to differing machine types or scanning parameters. Our results validate the claim of Haacke et al. (2005) that the RN is one of the tissues with a high iron concentration in the brain. Although the Ka of the RN of the PD group (Figure 1B) was lower than that of the HCs, only the difference between ESPD and HC was significant. We suspect that similar atrophic pathological changes occur in the RN and SN, which result in decreased microstructural complexity or heterogeneity in areas of maximal diffusion (Matuskey et al., 2020). In the RN, a multiple comparison correction for multiple correlation revealed no correlation between MS and MK, Ka, or Kr, either in the PD or HC groups (Figure 2B and Supplementary Figure 2B). This may mean that the damage associated with iron deposition in the RN is relatively mild compared to the SN and putamen and did not cause significant microstructure changes in the PD group.

### Striatum

Throughout neuronal degeneration, the number of major histocompatibility complex class II-positive microglia increased in both the SN and putamen (Imamura et al., 2003). A prospective study revealed MK elevation in the bilateral SN, putamen, GP, and caudate nucleus in patients with PD (Wang et al., 2011). In the putamen (Figure 1), only MS differences were observed in patients with PD versus HCs. However, positive correlations between MS and all three kurtosis indicators, especially, in the ASPD group were observed (Figures 2, 3). However, no correlations were observed between MS and MK, Ka, and Kr in the putamen of the HC group (Supplementary Figure 2C). This may further indicate the characteristic nature of the correlations in the putamen of the PD group. We hypothesize that since iron accumulation in PD is progressive, iron levels in the putamen had not accumulated sufficiently to cause microstructural differences between the groups. Pathological changes in the putamen correspond to Braak’s stage IV with low levels of iron deposition in the putamen in ESPD. Observed correlations were due to the characteristics of the ASPD group (Braak et al., 2003).

Autopsy results of GP iron levels are controversial. Our results and those of Chen et al. (1993) revealed increased levels of iron deposition, but levels reported by Riederer et al. (1989) were normal, and Dexter et al. (1991) observed a decrease. Differences may be due to the use of different procedures and quantification methods or measuring levels in the internal versus external GP (Griffiths et al., 1999). Changes in MK are associated with changes in myelin, axon, and neuronal density. Most current studies suggest that increased MK reflects glial cell proliferation or an increased density of myelin to cell ratio (Steven et al., 2014), and decreased MK may reflect histological changes in neuronal cell bodies or synapses, or mild demyelination changes (Gong et al., 2013). An interesting phenomenon was found in our results: PD compared to HCs showed opposite performances of MK for both SN and GP although MS was elevated for both (Figure 1). First, this may reflect a difference in pathological alterations because major histocompatibility complex class II -positive microglia proliferation occurs predominantly in the SN (Imamura et al., 2003). Second, this may be because of the different protein alterations in the SN and GP. Several amide proton transfer imaging studies have validated this possibility in terms of molecular imaging (Li et al., 2014a,2015a,^2017^). The team suggests that the main reason for the opposite signaling changes is that the SN is dominated by a reduction in dopaminergic neurons and dopamine production (Braak et al., 2003), whereas the GP is dominated by the deposition of cytoplasmic proteins and peptides (Tong et al., 2010). Finally, it is possible that axonal disintegration and cell loss play a dominant role in microstructural changes in the GP, and thus a decrease in MK. For example, two studies found a negative correlation between the MK of GP and age (Lätt et al., 2013; Gong et al., 2014). After multiple comparison correction of multiple correlations within each gray matter nucleus in the HC group, only correlations between MS and Ka in the caudate nucleus were found (Supplementary Figure 2E). We suggest that this may be due to systematic errors such as the small size of the caudate nucleus and the insufficient number of healthy controls included in this study. We will continue to increase the sample size in future studies to reduce the occurrence of such errors.

### Study Limitations

The study has some limitations. First, iron in the SN initially accumulates in the dorsal pars compacta; however, in the present study, the entire SN was assessed, which reduced sensitivity. Second, according to Braak’s stage, iron deposition initially occurs in the dorsal IX/X motor nucleus and/or intermediate reticular zone, and gradually accumulates in the coeruleus–subcoeruleus complex. By Braak’s stage III, a lesion forms in the SN, by which time clinical motor symptoms are already present. The present study assessed the correlation between iron deposition and tissue microstructure in the major gray matter nuclei. The association should be comprehensively studied in the future, considering all voxels from the medulla oblongata to the neocortex. Finally, most of the patients with PD included in the study were clinically symptomatic, with disease that was more severe than Braak stage III. It is not clear whether brain iron deposition in patients with PD with pre-Braak stage III disease will have a different kurtosis index profile. To address this problem, a study with a greater sample size, with preclinical patients with PD, is needed.

## Conclusion

In recent years, many efforts have been made to examine brain iron levels and their effects on patients with PD. Our study provides new insights into iron overload and associated microstructural alterations from a neuroimaging perspective, which has the potential to integrate previous findings. Tracking the dynamic changes in iron content and microstructure throughout the course of PD will help us to better understand the dynamics of iron metabolism and microstructural alterations in the pathogenesis of PD and to develop new approaches to monitor and treat PD. First, we found that iron deposition in the SN and putamen may have an impact on changes in brain microstructure in patients with PD. Increased SN iron deposition was positively correlated with MK and Kr, and negatively correlated with Ka. Increased iron deposition in the putamen was positively correlated with MK, Ka, and Kr. This was especially true for the SN in which correlations were observable during early-stage PD. In addition, magnetic sensitivity was significantly higher in patients with PD, especially in the SN, RN, putamen, and GP. Finally, we confirmed that iron deposition in the SN affects brain microstructure, and, potentially, motor function in PD.

## Data Availability Statement

The original contributions presented in the study are included in the article/Supplementary Material, further inquiries can be directed to the corresponding authors.

## Ethics Statement

The studies involving human participants were reviewed and approved by the Ethics Department of the Second Affiliated Hospital of Xiamen Medical College. The patients/participants provided their written informed consent to participate in this study.

## Author Contributions

LY, YC, YS, and YX: conceptualization, organization, and execution of research projects. JN, JG, YR, YJ, and ZZ: design, execution, and review of statistical analyses. LY, GY, and RW: wrote and reviewed the manuscript. All authors contributed to this article and approved the final submitted version.

## Conflict of Interest

The authors declare that the research was conducted in the absence of any commercial or financial relationships that could be construed as a potential conflict of interest.

## Publisher’s Note

All claims expressed in this article are solely those of the authors and do not necessarily represent those of their affiliated organizations, or those of the publisher, the editors and the reviewers. Any product that may be evaluated in this article, or claim that may be made by its manufacturer, is not guaranteed or endorsed by the publisher.

## Footnotes

1
https://cran.r-project.org

## References

[B1] Barnham K. J. Masters C. L. Bush A. I. (2004). Neurodegenerative diseases and oxidative stress. *Nat. Rev. Drug Discov.* 3 205–214. 10.1038/nrd1330 15031734

[B2] Batassini C. Broetto N. Tortorelli L. S. Borsoi M. Zanotto C. Galland F. (2015). Striatal Injury with 6-OHDA Transiently Increases Cerebrospinal GFAP and S100B. *Neural. Plast.* 2015:387028. 10.1155/2015/387028 26090233 PMC4451977

[B3] Bergsland N. Zivadinov R. Schweser F. Hagemeier J. Lichter D. Guttuso T. Jr. (2019). Ventral posterior substantia nigra iron increases over 3 years in Parkinson’s disease. *Mov. Disord* 34 1006–1013. 10.1002/mds.27730 31180615 PMC6642003

[B4] Block M. L. Zecca L. Hong J. S. (2007). Microglia-mediated neurotoxicity: uncovering the molecular mechanisms. *Nat. Rev. Neurosci.* 8 57–69. 10.1038/nrn2038 17180163

[B5] Braak H. Del Tredici K. Rüb U. de Vos R. A. Jansen Steur E. N. Braak E. (2003). Staging of brain pathology related to sporadic Parkinson’s disease. *Neurobiol. Aging* 24 197–211. 10.1016/s0197-4580(02)00065-9 12498954

[B6] Chen J. C. Hardy P. A. Kucharczyk W. Clauberg M. Joshi J. G. Vourlas A. (1993). MR of human postmortem brain tissue: correlative study between T2 and assays of iron and ferritin in Parkinson and Huntington disease. *AJNR Am. J. Neuroradiol.* 14 275–281. 8456699 PMC8332933

[B7] Cheung M. M. Hui E. S. Chan K. C. Helpern J. A. Qi L. Wu E. X. (2009). Does diffusion kurtosis imaging lead to better neural tissue characterization? A rodent brain maturation study. *Neuroimage* 45 386–392. 10.1016/j.neuroimage.2008.12.018 19150655

[B8] Coutu J. P. Chen J. J. Rosas H. D. Salat D. H. (2014). Non-Gaussian water diffusion in aging white matter. *Neurobiol. Aging* 35 1412–1421. 10.1016/j.neurobiolaging.2013.12.001 24378085 PMC3961541

[B9] Covell D. J. Robinson J. L. Akhtar R. S. Grossman M. Weintraub D. Bucklin H. M. (2017). Novel conformation-selective alpha-synuclein antibodies raised against different in vitro fibril forms show distinct patterns of Lewy pathology in Parkinson’s disease. *Neuropathol. Appl. Neurobiol.* 43 604–620. 10.1111/nan.12402 28386933 PMC5632188

[B10] Dexter D. T. Carayon A. Javoy-Agid F. Agid Y. Wells F. R. Daniel S. E. (1991). Alterations in the levels of iron, ferritin and other trace metals in Parkinson’s disease and other neurodegenerative diseases affecting the basal ganglia. *Brain* 114(Pt 4), 1953–1975. 10.1093/brain/114.4.1953 1832073

[B11] Dexter D. T. Wells F. R. Agid F. Agid Y. Lees A. J. Jenner P. (1987). Increased nigral iron content in postmortem parkinsonian brain. *Lancet* 2 1219–1220. 10.1016/s0140-6736(87)91361-4 2890848

[B12] Du G. Lewis M. M. Sica C. He L. Connor J. R. Kong L. (2018). Distinct progression pattern of susceptibility MRI in the substantia nigra of Parkinson’s patients. *Mov. Disord.* 33 1423–1431. 10.1002/mds.27318 29756399 PMC6185755

[B13] Du G. Liu T. Lewis M. M. Kong L. Wang Y. Connor J. (2016). Quantitative susceptibility mapping of the midbrain in Parkinson’s disease. *Mov. Disord.* 31 317–324. 10.1002/mds.26417 26362242 PMC5315570

[B14] Filli L. Wurnig M. Nanz D. Luechinger R. Kenkel D. Boss A. (2014). Whole-body diffusion kurtosis imaging: initial experience on non-Gaussian diffusion in various organs. *Invest. Radiol.* 49 773–778. 10.1097/rli.0000000000000082 24979203

[B15] Folstein M. F. Folstein S. E. McHugh P. R. (1975). Mini-mental state”. A practical method for grading the cognitive state of patients for the clinician. *J. Psychiatr. Res.* 12 189–198. 10.1016/0022-3956(75)90026-6 1202204

[B16] Ghosh D. Mehra S. Sahay S. Singh P. K. Maji S. K. (2017). α-synuclein aggregation and its modulation. *Int. J. Biol. Macromol.* 100 37–54. 10.1016/j.ijbiomac.2016.10.021 27737778

[B17] Giannelli M. Toschi N. Passamonti L. Mascalchi M. Diciotti S. Tessa C. (2012). Diffusion kurtosis and diffusion-tensor MR imaging in Parkinson disease. *Radiology* 265 645–646. 10.1148/radiol.12121036 23093710

[B18] Gong N. J. Wong C. S. Chan C. C. Leung L. M. Chu Y. C. (2013). Correlations between microstructural alterations and severity of cognitive deficiency in Alzheimer’s disease and mild cognitive impairment: a diffusional kurtosis imaging study. *Magn. Reson. Imag.* 31 688–694. 10.1016/j.mri.2012.10.027 23347602

[B19] Gong N. J. Wong C. S. Chan C. C. Leung L. M. Chu Y. C. (2014). Aging in deep gray matter and white matter revealed by diffusional kurtosis imaging. *Neurobiol. Aging* 35 2203–2216. 10.1016/j.neurobiolaging.2014.03.011 24910392

[B20] Greffard S. Verny M. Bonnet A. M. Beinis J. Y. Gallinari C. Meaume S. (2006). Motor score of the Unified Parkinson Disease Rating Scale as a good predictor of Lewy body-associated neuronal loss in the substantia nigra. *Arch. Neurol.* 63 584–588. 10.1001/archneur.63.4.584 16606773

[B21] Griffiths P. D. Dobson B. R. Jones G. R. Clarke D. T. (1999). Iron in the basal ganglia in Parkinson’s disease. An in vitro study using extended X-ray absorption fine structure and cryo-electron microscopy. *Brain* 122(Pt 4), 667–673. 10.1093/brain/122.4.667 10219780

[B22] Guan J. Ma X. Geng Y. Qi D. Shen Y. Shen Z. (2019). Diffusion Kurtosis Imaging for Detection of Early Brain Changes in Parkinson’s Disease. *Front. Neurol.* 10:1285. 10.3389/fneur.2019.01285 31920913 PMC6914993

[B23] Guan X. Xu X. Zhang M. (2017a). Region-Specific Iron Measured by MRI as a Biomarker for Parkinson’s Disease. *Neurosci. Bull.* 33 561–567. 10.1007/s12264-017-0138-x 28516282 PMC5636731

[B24] Guan X. Xuan M. Gu Q. Huang P. Liu C. Wang N. (2017b). Regionally progressive accumulation of iron in Parkinson’s disease as measured by quantitative susceptibility mapping. *NMR Biomed.* 30:4. 10.1002/nbm.3489 26853890 PMC4977211

[B25] Guan X. Xuan M. Gu Q. Xu X. Huang P. Wang N. (2017c). Influence of regional iron on the motor impairments of Parkinson’s disease: a quantitative susceptibility mapping study. *J. Magn. Reson. Imag.* 45 1335–1342. 10.1002/jmri.25434 27545971

[B26] Haacke E. M. Cheng N. Y. House M. J. Liu Q. Neelavalli J. Ogg R. J. (2005). Imaging iron stores in the brain using magnetic resonance imaging. *Magn. Reson. Imag.* 23 1–25. 10.1016/j.mri.2004.10.001 15733784

[B27] He N. Ling H. Ding B. Huang J. Zhang Y. Zhang Z. (2015). Region-specific disturbed iron distribution in early idiopathic Parkinson’s disease measured by quantitative susceptibility mapping. *Hum. Brain Mapp.* 36 4407–4420. 10.1002/hbm.22928 26249218 PMC6869507

[B28] Imamura K. Hishikawa N. Sawada M. Nagatsu T. Yoshida M. Hashizume Y. (2003). Distribution of major histocompatibility complex class II-positive microglia and cytokine profile of Parkinson’s disease brains. *Acta Neuropathol.* 106 518–526. 10.1007/s00401-003-0766-2 14513261

[B29] Jensen J. H. Helpern J. A. (2010). MRI quantification of non-Gaussian water diffusion by kurtosis analysis. *NMR Biomed.* 23 698–710. 10.1002/nbm.1518 20632416 PMC2997680

[B30] Jensen J. H. Helpern J. A. Ramani A. Lu H. Kaczynski K. (2005). Diffusional kurtosis imaging: the quantification of non-gaussian water diffusion by means of magnetic resonance imaging. *Magn. Reson. Med.* 53 1432–1440. 10.1002/mrm.20508 15906300

[B31] Jin L. Wang J. Zhao L. Jin H. Fei G. Zhang Y. (2011). Decreased serum ceruloplasmin levels characteristically aggravate nigral iron deposition in Parkinson’s disease. *Brain* 134(Pt 1), 50–58. 10.1093/brain/awq319 21109502

[B32] Kalia L. V. Lang A. E. (2015). Parkinson’s disease. *Lancet* 386 896–912. 10.1016/s0140-6736(14)61393-3 25904081

[B33] Kamagata K. Zalesky A. Hatano T. Ueda R. Di Biase M. A. Okuzumi A. (2017). Gray Matter Abnormalities in Idiopathic Parkinson’s Disease: evaluation by Diffusional Kurtosis Imaging and Neurite Orientation Dispersion and Density Imaging. *Hum. Brain Mapp.* 38 3704–3722. 10.1002/hbm.23628 28470878 PMC6867088

[B34] Kennedy P. R. Gibson A. R. Houk J. C. (1986). Functional and anatomic differentiation between parvicellular and magnocellular regions of red nucleus in the monkey. *Brain Res.* 364 124–136. 10.1016/0006-8993(86)90993-5 3947959

[B35] Khairnar A. Latta P. Drazanova E. Ruda-Kucerova J. Szabó N. Arab A. (2015). Diffusion Kurtosis Imaging Detects Microstructural Alterations in Brain of α-Synuclein Overexpressing Transgenic Mouse Model of Parkinson’s Disease: a Pilot Study. *Neurotox Res.* 28 281–289. 10.1007/s12640-015-9537-9 26153486

[B36] Koch J. C. Bitow F. Haack J. d’Hedouville Z. Zhang J. N. Tönges L. (2015). Alpha-Synuclein affects neurite morphology, autophagy, vesicle transport and axonal degeneration in CNS neurons. *Cell Death Dis.* 6:e1811. 10.1038/cddis.2015.169 26158517 PMC4650722

[B37] Kumar H. Lim H. W. More S. V. Kim B. W. Koppula S. Kim I. S. (2012). The role of free radicals in the aging brain and Parkinson’s Disease: convergence and parallelism. *Int. J. Mol. Sci.* 13 10478–10504. 10.3390/ijms130810478 22949875 PMC3431873

[B38] Landis J. R. Koch G. G. (1977). The measurement of observer agreement for categorical data. *Biometrics* 33 159–174. 843571

[B39] Langkammer C. Pirpamer L. Seiler S. Deistung A. Schweser F. Franthal S. (2016). Quantitative Susceptibility Mapping in Parkinson’s Disease. *PLoS One* 11:e0162460. 10.1371/journal.pone.0162460 27598250 PMC5012676

[B40] Lätt J. Nilsson M. Wirestam R. Ståhlberg F. Karlsson N. Johansson M. (2013). Regional values of diffusional kurtosis estimates in the healthy brain. *J. Magn. Reson. Imag.* 37 610–618. 10.1002/jmri.23857 23055442 PMC3596978

[B41] Li C. Chen M. Zhao X. Wang R. Chen H. Su W. (2017). Chemical Exchange Saturation Transfer MRI Signal Loss of the Substantia Nigra as an Imaging Biomarker to Evaluate the Diagnosis and Severity of Parkinson’s Disease. *Front. Neurosci.* 11:489. 10.3389/fnins.2017.00489 28912676 PMC5583514

[B42] Li C. Peng S. Wang R. Chen H. Su W. Zhao X. (2014a). Chemical exchange saturation transfer MR imaging of Parkinson’s disease at 3 Tesla. *Eur. Radiol.* 24 2631–2639. 10.1007/s00330-014-3241-7 25038850 PMC4471479

[B43] Li C. Wang R. Chen H. Su W. Li S. Zhao X. (2015a). Chemical Exchange Saturation Transfer MR Imaging is Superior to Diffusion-Tensor Imaging in the Diagnosis and Severity Evaluation of Parkinson’s Disease: a Study on Substantia Nigra and Striatum. *Front. Aging Neurosci.* 7:198. 10.3389/fnagi.2015.00198 26539109 PMC4609848

[B44] Li D. T. H. Hui E. S. Chan Q. Yao N. Chua S. E. McAlonan G. M. (2018). Quantitative susceptibility mapping as an indicator of subcortical and limbic iron abnormality in Parkinson’s disease with dementia. *Neuroimage Clin.* 20 365–373. 10.1016/j.nicl.2018.07.028 30128274 PMC6096006

[B45] Li W. Avram A. V. Wu B. Xiao X. Liu C. (2014b). Integrated Laplacian-based phase unwrapping and background phase removal for quantitative susceptibility mapping. *NMR Biomed.* 27 219–227. 10.1002/nbm.3056 24357120 PMC3947438

[B46] Li W. Wang N. Yu F. Han H. Cao W. Romero R. (2015b). A method for estimating and removing streaking artifacts in quantitative susceptibility mapping. *Neuroimage* 108 111–122. 10.1016/j.neuroimage.2014.12.043 25536496 PMC4406048

[B47] Martin W. R. Wieler M. Gee M. (2008). Midbrain iron content in early Parkinson disease: a potential biomarker of disease status. *Neurology* 70(16 Pt 2), 1411–1417. 10.1212/01.wnl.0000286384.31050.b5 18172063

[B48] Matuskey D. Tinaz S. Wilcox K. C. Naganawa M. Toyonaga T. Dias M. (2020). Synaptic Changes in Parkinson Disease Assessed with in vivo Imaging. *Ann. Neurol.* 87 329–338. 10.1002/ana.25682 31953875 PMC7065227

[B49] McGeer P. L. McGeer E. G. (2004). Inflammation and neurodegeneration in Parkinson’s disease. *Parkinsonism Relat. Disord.* 10 (Suppl. 1), S3–S7. 10.1016/j.parkreldis.2004.01.005 15109580

[B50] Mewes K. Cheney P. D. (1994). Primate rubromotoneuronal cells: parametric relations and contribution to wrist movement. *J. Neurophysiol.* 72 14–30. 10.1152/jn.1994.72.1.14 7965000

[B51] Millecamps S. Julien J. P. (2013). Axonal transport deficits and neurodegenerative diseases. *Nat. Rev. Neurosci.* 14:161–176. 10.1038/nrn3380 23361386

[B52] Onodera S. Hicks T. P. (2009). A comparative neuroanatomical study of the red nucleus of the cat, macaque and human. *PLoS One* 4:e6623. 10.1371/journal.pone.0006623 19675676 PMC2722087

[B53] Perlson E. Maday S. Fu M. M. Moughamian A. J. Holzbaur E. L. (2010). Retrograde axonal transport: pathways to cell death? *Trends Neurosci.* 33 335–344. 10.1016/j.tins.2010.03.006 20434225 PMC2902719

[B54] Pierpaoli C. Basser P. J. (1996). Toward a quantitative assessment of diffusion anisotropy. *Magn. Reson. Med.* 36 893–906. 10.1002/mrm.1910360612 8946355

[B55] Pinto T. C. C. Machado L. Bulgacov T. M. Rodrigues-Júnior A. L. Costa M. L. G. Ximenes R. C. C. (2019). Is the Montreal Cognitive Assessment (MoCA) screening superior to the Mini-Mental State Examination (MMSE) in the detection of mild cognitive impairment (MCI) and Alzheimer’s Disease (AD) in the elderly? *Int. Psychogeriatr.* 31 491–504. 10.1017/s1041610218001370 30426911

[B56] Riederer P. Sofic E. Rausch W. D. Schmidt B. Reynolds G. P. Jellinger K. (1989). Transition metals, ferritin, glutathione, and ascorbic acid in parkinsonian brains. *J. Neurochem.* 52 515–520. 10.1111/j.1471-4159.1989.tb09150.x 2911028

[B57] Rodriguez-Oroz M. C. Rodriguez M. Leiva C. Rodriguez-Palmero M. Nieto J. Garcia-Garcia D. (2008). Neuronal activity of the red nucleus in Parkinson’s disease. *Mov. Disord.* 23 908–911. 10.1002/mds.22000 18383534

[B58] Steven A. J. Zhuo J. Melhem E. R. (2014). Diffusion kurtosis imaging: an emerging technique for evaluating the microstructural environment of the brain. *AJR Am. J. Roentgenol.* 202 W26–W33. 10.2214/ajr.13.11365 24370162

[B59] Taylor K. I. Sambataro F. Boess F. Bertolino A. Dukart J. (2018). Progressive Decline in Gray and White Matter Integrity in de novo Parkinson’s Disease: an Analysis of Longitudinal Parkinson Progression Markers Initiative Diffusion Tensor Imaging Data. *Front. Aging Neurosci.* 10:318. 10.3389/fnagi.2018.00318 30349475 PMC6186956

[B60] Tong J. Wong H. Guttman M. Ang L. C. Forno L. S. Shimadzu M. (2010). Brain alpha-synuclein accumulation in multiple system atrophy, Parkinson’s disease and progressive supranuclear palsy: a comparative investigation. *Brain* 133(Pt 1), 172–188. 10.1093/brain/awp282 19903734

[B61] Uchida Y. Kan H. Sakurai K. Arai N. Kato D. Kawashima S. (2019). Voxel-based quantitative susceptibility mapping in Parkinson’s disease with mild cognitive impairment. *Mov. Disord* 34 1164–1173. 10.1002/mds.27717 31091347

[B62] Uchida Y. Kan H. Sakurai K. Inui S. Kobayashi S. Akagawa Y. (2020). Magnetic Susceptibility Associates With Dopaminergic Deficits and Cognition in Parkinson’s Disease. *Mov. Disord* 35 1396–1405. 10.1002/mds.28077 32369660

[B63] Wang J. J. Lin W. Y. Lu C. S. Weng Y. H. Ng S. H. Wang C. H. (2011). Parkinson disease: diagnostic utility of diffusion kurtosis imaging. *Radiology* 261 210–217. 10.1148/radiol.11102277 21771952

[B64] Weber R. A. Hui E. S. Jensen J. H. Nie X. Falangola M. F. Helpern J. A. (2015). Diffusional kurtosis and diffusion tensor imaging reveal different time-sensitive stroke-induced microstructural changes. *Stroke* 46 545–550. 10.1161/strokeaha.114.006782 25563646 PMC4418934

[B65] Wieler M. Gee M. Martin W. R. (2015). Longitudinal midbrain changes in early Parkinson’s disease: iron content estimated from R2*/MRI. *Parkinsonism Relat. Disord.* 21 179–183. 10.1016/j.parkreldis.2014.11.017 25534153

[B66] Wolozin B. Golts N. (2002). Iron and Parkinson’s disease. *Neuroscientist* 8 22–32. 10.1177/107385840200800107 11843096

[B67] Xu J. Xiao C. Song W. Cui X. Pan M. Wang Q. (2021). Elevated Heme Oxygenase-1 Correlates With Increased Brain Iron Deposition Measured by Quantitative Susceptibility Mapping and Decreased Hemoglobin in Patients With Parkinson’s Disease. *Front. Aging Neurosci.* 13:656626. 10.3389/fnagi.2021.656626 33815094 PMC8012799

[B68] Yang Z. Rong Y. Cao Z. Wu Y. Zhao X. Xie Q. (2021). Microstructural and Cerebral Blood Flow Abnormalities in Subjective Cognitive Decline Plus: diffusional Kurtosis Imaging and Three-Dimensional Arterial Spin Labeling Study. *Front. Aging Neurosci.* 13:625843. 10.3389/fnagi.2021.625843 33597860 PMC7882515

[B69] Zhang G. Zhang Y. Zhang C. Wang Y. Ma G. Nie K. (2015). Diffusion Kurtosis Imaging of Substantia Nigra Is a Sensitive Method for Early Diagnosis and Disease Evaluation in Parkinson’s Disease. *Parkinsons Dis.* 2015:207624. 10.1155/2015/207624 26770867 PMC4681830

[B70] Zhang W. Yan Z. F. Gao J. H. Sun L. Huang X. Y. Liu Z. (2014). Role and mechanism of microglial activation in iron-induced selective and progressive dopaminergic neurodegeneration. *Mol. Neurobiol.* 49 1153–1165. 10.1007/s12035-013-8586-4 24277523 PMC4878835

[B71] Zheng W. Wu C. Huang L. Wu R. (2017). Diffusion Kurtosis Imaging of Microstructural Alterations in the Brains of Paediatric Patients with Congenital Sensorineural Hearing Loss. *Sci. Rep.* 7:1543. 10.1038/s41598-017-01263-9 28484279 PMC5431550

