# Supplementary material for: Combined Application of Quantitative Susceptibility Mapping and Diffusion Kurtosis Imaging Techniques to Investigate the Effect of Iron Deposition on Microstructural Changes in the Brain in Parkinson’s Disease
Source: Front Aging Neurosci. 2022 Mar 15;14:792778. doi: 10.3389/fnagi.2022.792778 (PMC8965454; doi:10.3389/fnagi.2022.792778)
Supplement: Supplementary file 1 [file Data_Sheet_1.pdf]

Supplementary Material

1 Supplementary Table

Supplementary Table 1. Intraclass correlation coefficients (ICCs) analysis of QSM and DKI parameters in the left and right ROIs in normal controls and PD patients at different stages.

	**normal (ICCs)**		**ESPD (ICCs)**		**ASPD (ICCs)**	
	left	right	left	right	left	right
**MK**						
Substantia nigra	0.808	0.82	0.765	0.931	0.829	0.826
Red nucleus	0.777	0.885	0.768	0.756	0.767	0.775
Putamen	0.878	0.891	0.955	0.874	0.849	0.939
Globus pallidus	0.797	0.81	0.848	0.863	0.792	0.847
Caudate	0.915	0.946	0.775	0.863	0.881	0.902
**Ka**						
Substantia nigra	0.918	0.809	0.809	0.914	0.821	0.866
Red nucleus	0.814	0.905	0.960	0.945	0.913	0.865
Putamen	0.796	0.854	0.854	0.753	0.897	0.947
Globus pallidus	0.913	0.92	0.755	0.813	0.796	0.762
Caudate	0.936	0.956	0.887	0.891	0.935	0.902
**Kr**						
Substantia nigra	0.845	0.793	0.871	0.854	0.880	0.871

Red nucleus	0.903	0.923	0.785	0.843	0.838	0.915
Putamen	0.901	0.939	0.913	0.896	0.946	0.925
Globus pallidus	0.803	0.85	0.931	0.960	0.861	0.905
Caudate	0.921	0.918	0.938	0.886	0.943	0.924
**MS**						
Substantia nigra	0.785	0.871	0.940	0.932	0.755	0.866
Red nucleus	0.784	0.861	0.792	0.951	0.903	0.935
Putamen	0.824	0.89	0.861	0.768	0.752	0.855
Globus pallidus	0.936	0.93	0.800	0.831	0.877	0.782
Caudate	0.897	0.94	0.927	0.908	0.914	0.889

Abbreviations: ICCs, Intraclass correlation coefficients; ESPD, early-stage Parkinson's disease; ASPD, advanced-stage Parkinson's disease; MK, mean kurtosis; Ka, axial kurtosis; Kr, radial kurtosis; MS, magnetic susceptibility

Supplementary Table 2. Measured MK、 Ka、 Kr and MS values (mean±95 % CI) for the substantia nigra, red nucleus, globus pallidus, putamen and caudate for normal controls and PD patients at different stages. (FDR-corrected)

	**normal (N= 25)**	**ESPD (N= 24)**	**ASPD (N = 13)**	***p* value**
**MK**				
Substantia nigra	1.08±0.24	1.26±0.35	1.44±0.18	**<0.001*****, **<0.001*****,0.51
Red nucleus	1.14±0.26	1.12±0.18	1.10±0.14	0.55,0.55,0.55
Putamen	0.74±0.14	0.69±0.20	0.74±0.20	0.12,0.83,0.12
Globus pallidus	0.98±0.18	0.89±0.20	0.90±0.20	**0.004****,**0.02***,0.78
Caudate	0.65±0.16	0.60±0.20	0.66±0.18	0.16, 0.85,0.16
**Ka**				
Substantia nigra	0.80±0.22	0.58±0.12	0.56±0.08	**<0.001*****, **<0.001*****,0.33
Red nucleus	0.90±0.14	0.72±0.31	0.80±0.29	**<0.001*****,0.10,0.11
Putamen	0.89±0.16	0.84±0.20	0.91±0.31	0.28, 0.61,0.28
Globus pallidus	0.97±0.24	1.01±0.18	1.02±0.27	0.19,0.19, 0.75
Caudate	0.73±0.20	0.69±0.20	0.74±0.16	0.20, 0.82,0.20
**Kr**				
Substantia nigra	1.40±0.55	2.18±0.76	2.35±0.29	**<0.001*****, **<0.001*****,0.24
Red nucleus	1.40±0.47	1.46±0.43	1.37±0.39	0.57,0.73,0.57
Putamen	0.66±0.14	0.60±0.24	0.63±0.24	0.08,0.44,0.44

Globus pallidus	1.04±0.27	0.79±0.26	0.79±0.26	**<0.001*****, **<0.001*****,0.95
Caudate	0.67±0.24	0.58±0.20	0.59±0.22	**0.02***,0.08,0.70
**MS**				
Substantia nigra	0.10±0.06	0.14±0.10	0.17±0.10	**0.003****, **<0.001*****,**0.05***
Red nucleus	0.09±0.08	0.13±0.10	0.15±0.10	**0.004****,**0.001****,0.30
Putamen	0.08±0.04	0.09±0.03	0.11±0.04	**0.02***, **<0.001*****,**0.001****
Globus pallidus	0.12±0.06	0.16±0.08	0.17±0.08	**<0.001*****,**<0.001*****,0.44
Caudate	0.04±0.02	0.04±0.04	0.05±0.02	0.33,0.18,0.39

MK、 Ka and Kr are dimensionless, and magnetic susceptibility (MS) is expressed in parts per million (ppm). *p* value (first: normal vs ESPD; second: normal vs ASPD; third: ESPD vs ASPD); Bold: Significant change. Abbreviations: ESPD, early-stage Parkinson's disease; ASPD, advanced-stage Parkinson's disease; MK, mean kurtosis; Ka, axial kurtosis; Kr, radial kurtosis; MS, magnetic susceptibility.

2 Supplementary Figures

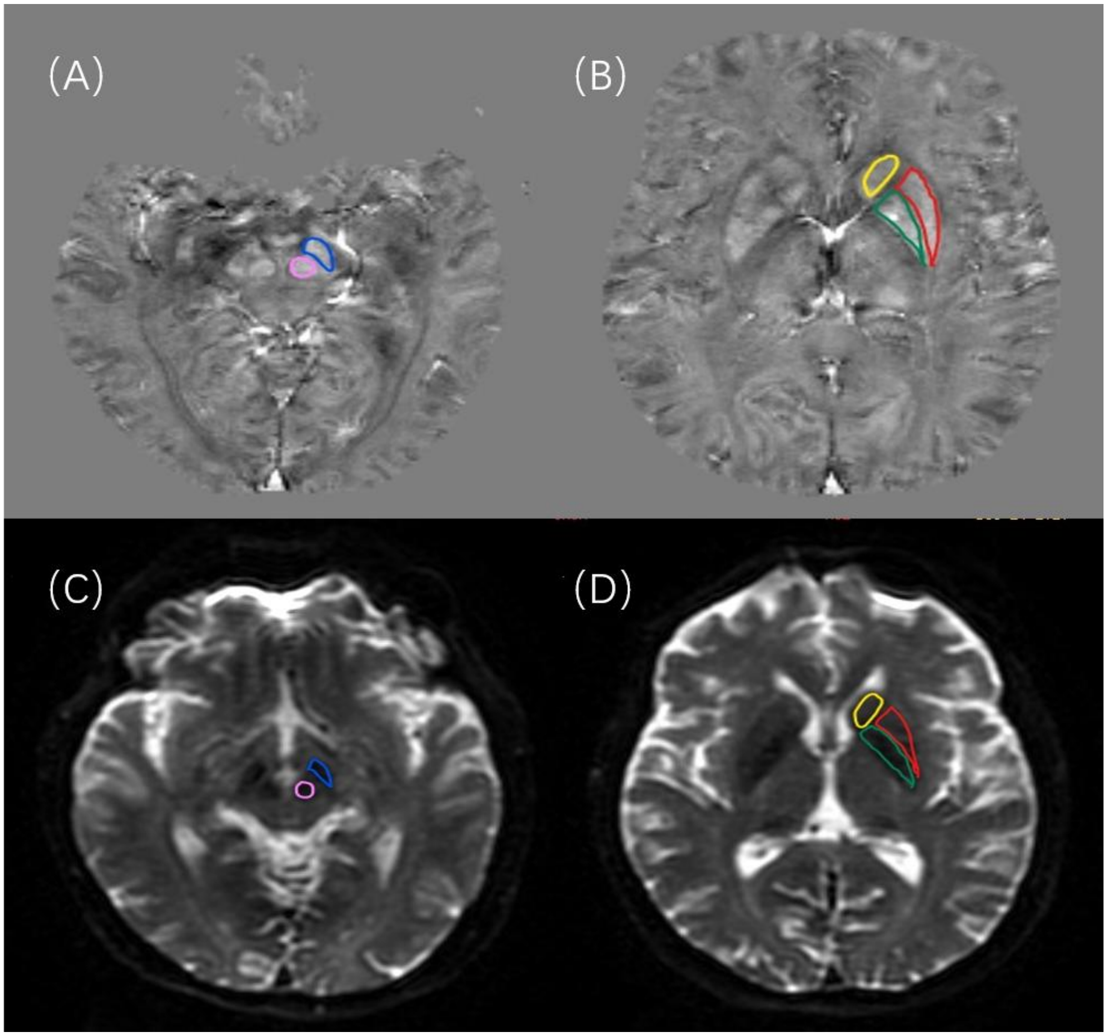

Supplementary Figure 1. The regions of interest (ROIs) used for quantitative analysis were manually drawn for quantitative susceptibility mapping (a, b) and B0 map (c, d) for a 57-year-old healthy man. All maps for ROIs in the basal ganglia displayed high degrees of contrast relative to surrounding tissue. Magnetic susceptibility (MS), mean kurtosis (MK), axial kurtosis (Ka)， and radial kurtosis (Kr) were measured for each region, and values of each side were recorded as separate samples. The substantia nigra (blue); red nucleus (pink); globus pallidus (green); putamen (red); and caudate (yellow) are shown.

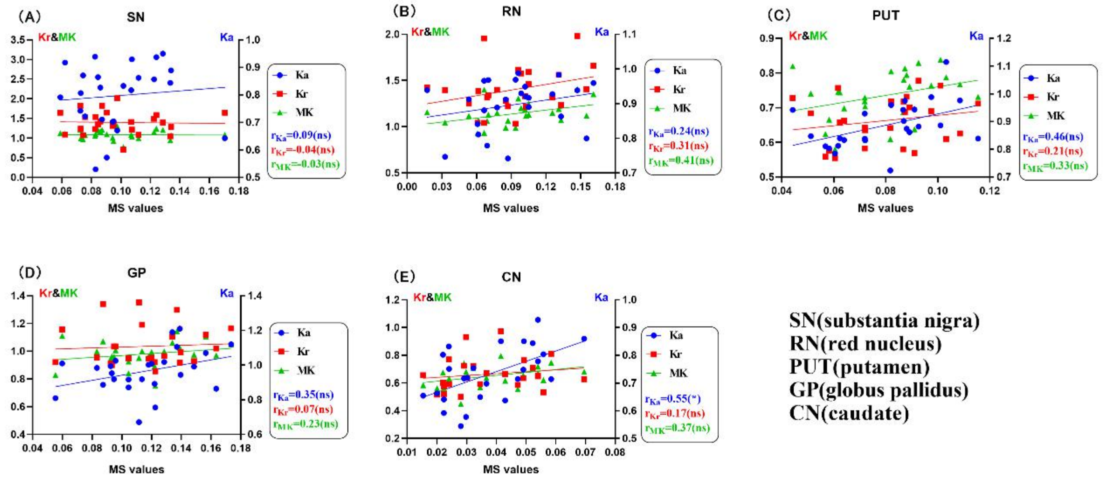

Supplementary Figure 2. Univariate correlations of diffusion kurtosis parameters with magnetic susceptibility values for healthy controls in five nucleus of interest (*P < 0.05; ns, no statistical significance). False discovery rate correction was used for multiple correlations. Abbreviations: MK (green, triangles), mean kurtosis; Ka (blue, circles), axial kurtosis; Kr (red, squares), radial kurtosis; MS, magnetic susceptibility.

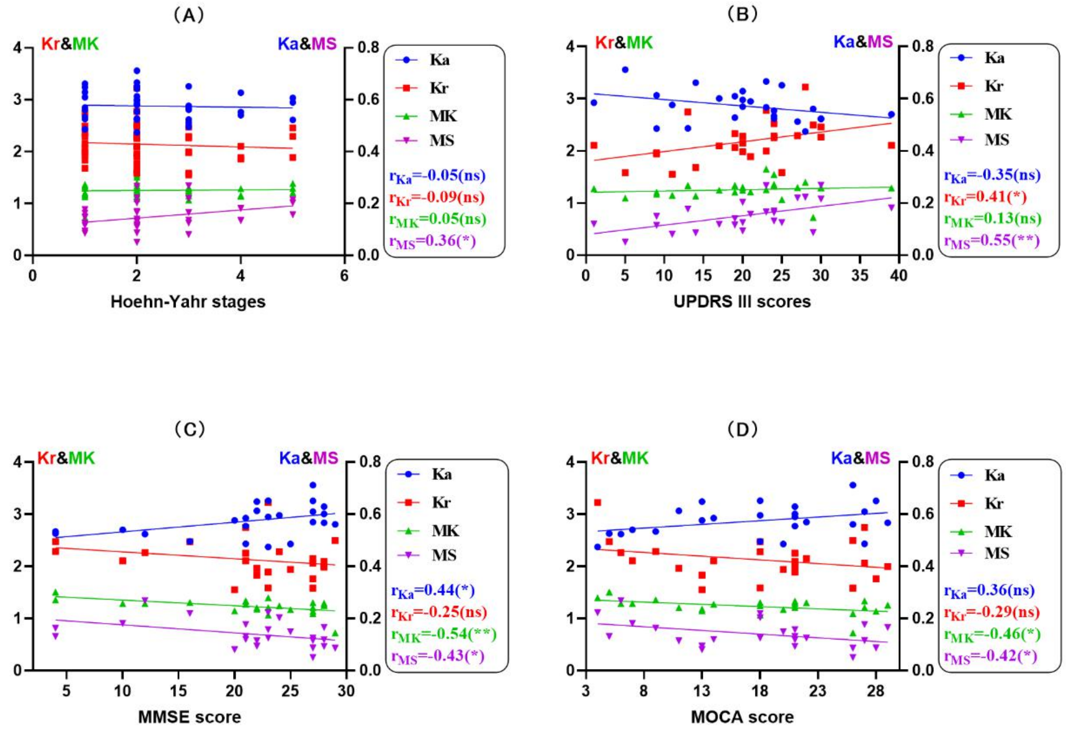

Supplementary Figure 3. Correlation between quantitative susceptibility mapping and diffusion kurtosis imaging metrics and clinical indices in the substantia nigra of patients with Parkinson’s disease (*P < 0.05; **P < 0.01; ns, no statistical significance). Abbreviations: UPDRS, Unified Parkinson's Disease Rating Scale; MMSE, Mini-mental State Examination; MoCA, Montreal Cognitive Assessment Scale; MK (green, triangles), mean kurtosis; Ka (blue, circles), axial kurtosis; Kr (red, squares), radial kurtosis; MS (purple, inverted triangles), magnetic susceptibility.
